# Review of current literature on gestational trophoblastic neoplasia

**DOI:** 10.1186/s43046-023-00195-y

**Published:** 2023-11-27

**Authors:** Mehwish Shahzadi, Saqib Raza Khan, Muhammad Tariq, Sehrish Sarwar Baloch, Aisha Shahid, Munira Moosajee, Zarka Samon

**Affiliations:** 1 Department of Medical Oncology, Aga Khan University Hospital, Karachi, Pakistan; 2 Department of Medical Oncology, Khyber Teaching Hospital, Peshawar, Pakistan; 3 Department of internal medicine, Jinnah Postgraduate Medical Center, Karachi, Pakistan; 4 Department of Oncology, Monash Health, Bentleigh East, Australia

**Keywords:** Bleeding, chemo-resistant clone, fertilization, methotrexate

## Abstract

**Background:**

Gestational Trophoblastic Neoplasia (GTN) is a disease of the reproductive age group with an incidence rate of <1% among all tumors involving the female reproductive tract. It occurs because of aberrant fertilization. Patients are diagnosed early because of aggravated symptoms during pregnancy. Moreover, patients also bleed from the tumor sites, which leads to early presentation. A cure rate of 100% can be achieved with adequate treatment.

**Main body:**

In this literature review, the authors have brought to attention the risk factors, classification, and various treatment options in GTN patients according to their stratification as per the WHO scoring system. Patients are categorized into low and high risk based on the FIGO scoring system. Patients with low risk are treated with single-agent methotrexate or actinomycin-D. Despite the superiority of actinomycin-D in terms of efficacy, methotrexate remains the first choice of therapy in low-risk patients due to its better toxicity profile. Multi-agent chemotherapy with etoposide, methotrexate, actinomycin-D, cyclophosphamide and vincristine (EMA-CO) leads to complete remission in 93% of high-risk GTN patients. Around 40% of patients with incomplete responses are salvaged with platinum-based multi-agent chemotherapy. Isolated chemo-resistant clones can be salvaged with surgical interventions.

**Conclusion:**

The mortality in patients with GTN has significantly reduced over time. With adequate multi-disciplinary support, patients with GTN can ultimately be cured and can spend every day healthy reproductive life.

## Introduction

Gestational Trophoblastic Neoplasia (GTN) is a rare disease with an incidence rate of <1% among all tumors involving the female reproductive tract [1]. There is a 90 – 100% cure rate for this disease, even with the presence of metastasis [2, 3]. GTN is the malignant form of Gestational Trophoblastic Disease (GTD) which includes a group of tumors that arise from the abnormal proliferation of the trophoblast of the placenta [4]. The GTD has various histological subtypes. They are broadly classified into benign, non-neoplastic trophoblastic lesions, hydatiform moles and GTN. Exaggerated placental site (EPS) reaction and placental site nodules are frequent benign lesions incidentally diagnosed after endometrial curettage or hysterectomy. These lesions lack necrosis with little to no mitotic activity. Hydatidiform mole is the most common histological form of GTD, comprising 80% of the cases [4, 5]. Although they are benign, they can increase the risk of malignant GTN. It comprises a complete hydatiform mole and a partial hydatiform mole. The invasive mole accounts for 15% of patients. The true GTN includes choriocarcinoma, placental site trophoblastic tumor (PSTT) and epithelioid trophoblastic tumor (ETT), which comprises the remaining 5% of cases. The World Health Organization (WHO) adds abnormal-non molar villous lesions as an additional category, mimicking the histological features of the partial hydatiform mole [6–8]. DNA genotyping may help to distinguish these forms from partial hydatiform moles. The differential diagnosis of GTN includes ectopic pregnancy, incomplete abortion, cornual pregnancy, and HCG-secreting germ cell tumors. Therefore, detailed pathological findings and clinical correlation are essential to diagnose accurately.

Epidemiological studies have reported wide regional variations in the incidence of GTN [9]. For example, in the western world, its incidence is reported to be one out of every 1000 pregnancies, while in third-world countries, it is as high as two out of every 1000 pregnancies [10]. This difference could be related to the dietary deficiency of vitamin A, which poses a risk of molar pregnancy [11].

GTN results from aberrant fertilization. However, it is primarily the over-expression of paternal genes which results in the malignant potential of GTN [12]. This was notified by John R Davis in 1984 when he found the Y chromosome in 9% of hydatidiform moles, 50% of invasive moles and 74% of choriocarcinoma patients [12]. Apart from this, many genetic mutations have been identified as a potential for the development of GTN [13]. These include mutation in p53, p21, Rb, c-myc, c-erb-3, MDM-2, and EGFR over-expression [14]. Since there is a lack of activating mutations in the tyrosine kinase domain of EGFR, conventional anti-EGFR therapies have no role in GTN management [15].

There are also some risk factors which lead to increased risk of GTN. These include an increasing maternal age of around 40, previous history of molar pregnancy, blood group A and Asian ancestry [16]. Pregnancy in a teenager also poses an increased risk for GTN [16]. Despite being a disease of the reproductive age group, patients with GTN, if treated appropriately, have retained fertility [17]. Literature shows various case reports in which GTN survivors delivered an average healthy child [18].

GTN patients seek medical advice because of a wide range of clinical symptoms [19]. This usually depends on the site and the extent of disease involvement. Patients mainly present with bleeding, which could be either from the primary site, that is, the uterus, in the form of irregular vaginal bleeding [20], or it could be from the metastatic site, like the lungs, in the form of hemoptysis [21]. This is because of the fragile blood vessels in abnormally proliferating trophoblastic cells [22]. Raised levels of human chorionic gonadotrophin (hCG) (>100,000 m IU/ml) may cause symptoms of hyperthyroidism, hyperemesis, pre-eclampsia and rarely virilization by ovarian theca lutein cyst formation [19, 23]. The patient also presents specific symptoms of the organ involved, like shortness of breath, cough, chest pain, deranged liver function test and unexplained drowsiness. Therefore, checking human chorionic gonadotrophin levels in all female patients with multi-organ involvement is highly recommended, as GTN can have an unusual presentation [2, 24].

### Risk stratification

Patients with GTN are divided into two broad categories - low-risk and high-risk [25]. This stratification is based on the International Federation of Gynecology and Obstetrics (FIGO) staging (Table 1) and World Health Organization (WHO) risk scoring systems (Table 2). The FIGO staging system applies to invasive mole, choriocarcinoma and PSTT.

**Table 1:** FIGO staging system

Figo^**a**^ staging for gestational trophoblastic neoplasia	
Stage 1: Lesion confined to the uterus	
Stage 2: Lesion extends outside the uterus but is limited to the genital structures (adnexa, vagina, broad ligament)	
Stage 3: Lesions are seen in the lungs	
Stage 4: All other metastatic sites.	

^a^(FIGO: International Federation of Gynecology and Obstetrics)

**Table 2:** Modified WHO prognostic scoring system

Risk Factor	0	1	2	4
Age (Years)	<40	>40	-	-
Antecedent pregnancy	Mole	Abortion	Term	-
The interval from last pregnancy (months)	4	4 to 6	7 to 12	>12
Pre-treatment serum hCG (m IU/mL)	<1000	1000 to 10000	10,000 till 100000	>100000
Largest tumor size	<3cm	3 to 4 cm	>5 cm	-
Site of metastasis	Lung	Spleen, Kidney	GI tract	Brain, Liver
No of metastasis	-	1 to 4	5 to 8	>8
Prior failed chemotherapy	-	-	1 drug	≥2 drugs

WHO Prognostic scoring system includes variables like age, antecedent pregnancy, the interval from last pregnancy, pre-treatment serum beta-human chorionic gonadotrophin levels, size of the largest lesion, number of metastatic lesions, site of metastasis and usage of any prior chemotherapy regimen [26]. A score of less than seven is considered a low-risk disease, while a score of seven or above is high-risk and requires urgent treatment with multi-agent chemotherapy [27].

### Management

#### Low-risk disease

Treatment of low-risk GTN comprises single-agent chemotherapy [28]. The two best drugs to date are dactinomycin and methotrexate [29]. The efficacy and toxicities of these drugs have been evaluated in various trials, and both successfully achieved complete remission in patients with GTN [30, 31]. In terms of efficacy, dactinomycin was superior to methotrexate, as shown in a study by Ruifang-An et al. In this study, the investigator reported a complete remission rate of 80% vs 65% in the dactinomycin and methotrexate groups, respectively [31]. Similarly, in a phase III study by Osborne et al., a response rate of 73% vs 58% was reported for pulsed dactinomycin vs weekly scheduled methotrexate [32]. The tolerance to therapy was similar in both groups. Yarandi F et al. also reported similar results. In this study, a pulse dose of intramuscular methotrexate (30 mg/m2) was given to 80 patients, and another 50 patients received an intravenous bolus of dactinomycin (1.25 mg/m2) every two weeks. Like previous studies, this also demonstrated the superiority of dactinomycin in terms of achieving complete remissions (90% vs 48%) in GTN patients (p <0.001) [33]. Moreover, the number of cycles used in treatment was also less in dactinomycin than in methotrexate [33].

Despite dactinomycin being more efficacious, methotrexate is still preferentially used for treating low-risk GTN [34]. One reason is the favorable toxicity profile of methotrexate, especially concerning alopecia which is complete with dactinomycin [35]. Considering the age group of patients with GTN, getting complete alopecia with a drug is a significant obstacle to treatment; hence patients choose methotrexate over dactinomycin despite its superiority in terms of efficacy [36]. Newer dosing schedule of methotrexate which was given as a five-day regimen at a dose of 0.4 mg/kg/day (maximum 25mg/day), cured 226 patients out of 253 (89.3%) in Brewer Trophoblastic Disease Centre (USA) as shown by Lurain JR et al. in his study [37]. This showed a response rate of 89%. Hence a suitable adverse profile and an overall acceptable response rate make methotrexate a treatment of choice for patients with low-risk GTN.

Methotrexate might not be the drug of choice for all patients. Several factors are associated with the resistance to single-agent methotrexate. These include human chorionic gonadotropin level >50,000 m IU/ml, non-molar antecedent pregnancy and clinicopathologic diagnosis of choriocarcinoma [37]. In such cases, multi-agent chemotherapy, including methotrexate, cure the patients [38]. Additionally, patients with deranged liver function tests and those with effusions are also not ideal candidates for treatment with methotrexate [35].

#### High-risk disease

High-risk GTN includes Stage II and III patients with FIGO score > seven and patients with metastatic disease on presentation. Lung, liver, and brain are the most common metastatic sites in GTN [39]. Being a chemo-sensitive disease, combining chemotherapy is the treatment of choice [29]. For the last two decades, multi-agent chemotherapy using etoposide, methotrexate, actinomycin-D, cyclophosphamide and vincristine (EMA-CO) remains the standard of care treatment for patients with high-risk GTN [40]. It has shown a remarkable response rate of 93%, plus a decrease in mortality rate to just 9%, which was previously reported to be around 30% with MA, CHAMOCA and MAC [41]. Therefore, EMA-CO is considered a standard of care for patients with high-risk GTN.

About 11% of GTN patients have brain metastasis on presentation [42]. This can be life-threatening as these vascular deposits can result in intracranial haemorrhage plus devastating neurological deficit. For treating such patients, the same regimen of EMA-CO but with a higher dose and infusion rate of methotrexate (1000mg/m2 given over 12 to 24 hours) is administered. This allows an adequate dose of methotrexate within the cerebrospinal fluid (CSF) which provides a complete response in patients negating the need for whole brain radiation [41, 43]. This improves the five years’ survival to 81.5%, with 75% of the patients resuming normal life [44]. Poor prognostic factors in patients with brain metastasis include the age of more than 40 years, presence of concomitant renal metastasis, FIGO score of over 12, and previous history of failure with multidrug chemotherapy [2].

Patients with GTN with widespread metastasis other than the lung and vagina and a high prognostic score (>12) are at high risk of intra-cranial, pulmonary, and intra-peritoneal haemorrhage. To minimize this risk, a short course of weekly etoposide (100mg/m2 D1+D2) and cisplatin (20mg/m2 D1+D2) should be given prior to EMA-CO. Approximately 30 – 40% of patients developed increased beta HCG levels post-completion of treatment with EMA-CO [45]. Such patients can be salvaged with EMA-EP, which shows a response rate of 84.9% [46]. Patients who develop methotrexate resistance, i.e., have also progressed on EMA-EP, can be treated with a combination of paclitaxel and cisplatin weekly alternating with paclitaxel and etoposide (TP/TE) [47]. Various chemotherapy combinations with activity in germ cell tumors (e.g., BEP, VIP, TIP) are also being used in this methotrexate resistant group with a success rate of about 80% [48]. Moreover, adjuvant surgical procedures can be employed to treat chemotherapy-resistant isolated tumor sites like the lungs and brain [49, 50] (Table 3).

**Table 3:** Treatments and remission rates in low-risk and high-risk gestational trophoblastic neoplasia

Reference	Year of Trial	Treatment arms	Complete Remission (CR) Rate	P VALUE	95% CI
**Low-Risk GTN**					
Hao J et al.	2021	Actinomycin vs Methotrexate	80.20% vs 65.1%	0.103	1.70 – 2.73
Osborne et al.	2011	Dactinomycin vs Methotrexate	70% vs 53%	0.03	-----
Yarandi F et al.	2008	Actinomycin vs Methotrexate	90.00% vs 48.10%	<0.001	5.7 – 22.6
Aghajanian C et al.	2011	Dactinomycin vs Methotrexate	70% vs 53%		
**High-Risk GTN**					
May T et al.	2011	EMA-CO vs MFA vs MAC vs CHAMOCA	91% vs 63% vs 68% vs 71%		
Lu WG et al.	2008	EMA-CO	77.8%		
Matsui H et al.	2004	MEA vs FA	69.7% vs 81.8%		
Aminimoghaddam S et al.	2018	EMA-EP	88%		

*MFA* Methotrexate, folinic acid, ACT-D; *CHAMOCA* Cyclophosphamide, hydroxycarbamide, doxorubicin, ACT-D, Methotrexate, melphalan and Vincristine; *MEA* Methotrexate, Etoposide and Actinomycin D; *FA* 5-Fluorouracil and Actinomycin D

### Role of immunotherapy

Almost all GTN tumor cells express programmed cell death ligand 1 (PD-L1) [51]. This led to the testing of PD-L1 antibodies in GTN patients. Current studies have suggested high expressions of PDL-1 in the normal placenta as well as on various histological subtypes of GTD [51, 52]. Bolze PA et al. analyzed the level of PDL-1 expression in all forms of GTN. They demonstrated a PDL-1 positivity of 80% in the specimens of choriocarcinoma [5, 8, 50–57]. In a phase II TROPHIMMUN trial, avelumab, a monoclonal antibody targeting PD-L1, is used in low-risk GTN patients who have disease progression after single-agent chemotherapy (methotrexate or dactinomycin). In this study, avelumab showed a favourable safety profile and a CR rate of 53.3% [53]. In various case reports, pembrolizumab has also demonstrated a response in chemotherapy-refractory GTN patients [54–56]. This makes immune checkpoint inhibitors a possible salvage option in GTN patients. However, this needs further evaluation through extensive studies.

## Conclusion

With the current multi-disciplinary treatment modalities, mortality in patients with GTN has significantly reduced. The chemotherapy-resistant disease is the major reason for GTN patients' mortality, while in the past, it was the bleeding from the metastatic site. We hope that with ongoing research and better treatment protocols, we might achieve excellent survival in patients with GTN.

## Data Availability

Not applicable

## Abbreviations

GTN: Gestational Trophoblastic Neoplasia
WHO: World Health Organization
PSTT: Placental site trophoblastic tumor.
ETT: Placental site trophoblastic tumor.
CSF: Cerebrospinal fluid
